# Common-path multimodal three-dimensional fluorescence and phase imaging system

**DOI:** 10.1117/1.JBO.25.3.032010

**Published:** 2020-02-06

**Authors:** Manoj Kumar, Xiangyu Quan, Yasuhiro Awatsuji, Chaoyang Cheng, Mitsuyasu Hasebe, Yosuke Tamada, Osamu Matoba

**Affiliations:** a Kobe University, Graduate School of System Informatics, Kobe, Japan; b Kyoto Institute of Technology, Faculty of Electrical Engineering and Electronics, Kyoto, Japan; c National Institute for Basic Biology, Okazaki, Japan; d ERATO, JST, Okazaki, Japan; e SOKENDAI (The Graduate University for Advanced Studies), School of Life Science, Okazaki, Japan; f Utsunomiya University, School of Engineering, Utsunomiya, Japan

**Keywords:** multimodal, common-path configuration, digital holography, three-dimensional fluorescence imaging, three-dimensional phase imaging

## Abstract

A stable multimodal system is developed by combining two common-path digital holographic microscopes (DHMs): coherent and incoherent, for simultaneous recording and retrieval of three-dimensional (3-D) phase and 3-D fluorescence imaging (FI), respectively, of a biological specimen. The 3-D FI is realized by a single-shot common-path off-axis fluorescent DHM developed recently by our group. In addition, we accomplish, the phase imaging by another single-shot, highly stable common-path off-axis DHM based on a beam splitter. In this DHM configuration, a beam splitter is used to divide the incoming object beam into two beams. One beam serves as the object beam carrying the useful information of the object under study, whereas another beam is spatially filtered at its Fourier plane by using a pinhole and it serves as a reference beam. This DHM setup, owing to a common-path geometry, is less vibration-sensitive and compact, having a similar field of view but with high temporal phase stability in comparison to a two-beam Mach–Zehnder-type DHM. The performance of the proposed common-path DHM and the multimodal system is verified by conducting various experiments on fluorescent microspheres and fluorescent protein-labeled living cells of the moss *Physcomitrella patens*. Moreover, the potential capability of the proposed multimodal system for 3-D live fluorescence and phase imaging of the fluorescent beads is also demonstrated. The obtained experimental results corroborate the feasibility of the proposed multimodal system and indicate its potential applications for the analysis of functional and structural behaviors of a biological specimen and enhancement of the understanding of physiological mechanisms and various biological diseases.

## 1 Introduction

Various multimodal imaging systems with different configurations and for different purposes have been recently developed because these systems enable one to analyze the functional and structural behaviors of a biological specimen at a single examination and, therefore, facilitate a better understanding of the behavior of molecular, cellular, and disease biology.^1^^–^^9^ The multimodal systems were developed for simultaneous fluorescence and quantitative phase imaging by incorporating the two-dimensional (2-D) epifluorescence microscopy with the diffraction phase microscopy by Park et al.^1^ and with the Mach–Zehnder-type digital holographic microscopy (DHM) by Pavillon et al.^2^ and Quan et al.^3^ A multimodal approach that incorporates confocal Raman, confocal reflectance, and quantitative phase microscopy^4^ has demonstrated the potential for retrieving the molecular specific and morphological information. Optical diffraction tomography (ODT) is another technique to reconstruct the three-dimensional (3-D) quantitative phase imaging from the recorded multiple 2-D holograms. This technique was exploited with the optofluidic rotation of suspended cells by Schürmann et al.^5^ to measure the 3-D correlated refractive index and fluorescence data for a quantitative interpretation of the nuclear refractive index. Further, a multimodal system was developed by integrating the ODT and 3-D structured illumination microscopy by Shin et al.^6^ for the measurement of 3-D refractive index and fluorescence distribution of live cells. The structured illumination microscopy-based multimodal system^7^ is demonstrated for 3-D subdiffraction multimodal imaging of both quantitative phase and fluorescence. Chowdhury et al.^8^ and Nygate et al.^9^ proposed the multimodal systems based on the principle of off-axis holographic multiplexing to obtain the quantitative phase and fluorescence imaging (FI) of the biological cells using a single camera. These systems,^8^^,^^9^ owing to the use of single camera for recording both the phase hologram and the fluorescence image, are free from image registration issues.

A multimodal system combining the DHM with the FI may address several critical concerns of biology. The DHM system concedes the structural information by exploiting the optical path-length shifts through the biological specimen, and the FI reveals the functional details of the specific molecules of interest in the specimen. Thus, a multimodal system comprising a DHM and an FI provides two distinct information of the specimen simultaneously through a single examination. Our group previously proposed a multimodal system in which phase imaging is realized by Mach–Zehnder-type DHM.^3^ However, the phase measurement is less stable due to the two-arm configuration of DHM. Furthermore, it is not easy to make it compact in the optical setup. For the time-lapse measurement of live cell imaging in biological applications, in this paper, we propose a single-shot common-path off-axis DHM based on a beam splitter. Then, we developed a multimodal system comprising the proposed DHM and another single-shot common-path off-axis fluorescent digital holographic microscope (FDHM), which was recently developed by our group.^10^ The proposed multimodal system enables one to provide a stable system of 3-D phase and 3-D FI of a biological specimen in a common hybrid platform.

The 3-D fluorescence microscopy has received significant interest since it is becoming a crucial tool in molecular and cellular biology to understand the dynamics of specific molecules, organelles, and structures in a biological sample. Several techniques have been used in high-resolution 3-D FI such as confocal,^11^ two-photon,^12^ or light sheet microscopy.^13^ However, these methods are inherently limited in terms of speed or volume, because they all involve scanning. Light field microscopy^14^^,^^15^ achieves single-shot 3-D capture but sacrifices resolution. Coded aperture microscopy^16^^–^^19^ provides high-resolution imaging with single-shot capture, but it requires an extremely sparse sample. On the other hand, continuous progress in the field of incoherent holography opens new possibilities of 3-D FI. Digital holography can be adopted to fluorescence microscopy for recording and retrieving the 3-D information of incoherent fluorescent objects.^20^^–^^24^ Our group reported a fluorescent digital holographic system by using a dual-focusing lens with a diffraction grating.^23^ In this configuration, the 0-th order unmodulated light is cut out and the interference occurs between the two first orders. However, the major limitation of this configuration is that the utilization factor of the light of the first orders becomes very low. The aforementioned limitation of the incoherent digital holographic system is resolved by proposing a modified configuration of the common-path off-axis incoherent digital holographic system^10^ for the 3-D FI of biological samples.

The 3-D phase imaging techniques provide morphological information about the structure and dynamics of the transparent specimen.^25^^,^^26^ An off-axis DHM is an ideal tool for studying various biological parameters, including 3-D imaging and parameter extraction, and for the measurement of 3-D profiling and tracking,^27^ refractive index,^28^ spectral dispersion,^29^ dry mass localization,^30^ and optimum focus determinations.^31^ Moreover, the technique has been employed for monitoring cell growth, characterizing cellular motility, and investigating the subcellular motions of living cells.^32^ The DHM systems usually employ the two-beam Mach–Zehnder interferometric configuration for quantifying the cellular and subcellular structures. In this geometry, two beams, following separate paths and passing through separate sets of optical components, are used to form the interference pattern on the image sensor. These separate paths for the object and reference beams may lead to lower spatial and temporal phase stability,^33^ limitation on measuring small cell thickness fluctuation,^34^ and complex and costly system setup. These complications can be overcome by employing a common-path configuration.^35^^–^^43^ In common-path DHM systems, the object and reference beams follow approximately the same path and, therefore, make the systems less prone to environmental vibrations/perturbations. However, in such systems, both beams carry identical information and have enormous chances of overlapping at the image sensor. To overcome this problem, self-referencing techniques, in which the reference beam consists of only a small portion of the object beam, which does not carry object information, are proposed.^35^^,^^38^^,^^44^ Also, approaches to employing a pinhole to one of the beams at its Fourier plane in order to erase all the object information in one beam and it is serving as a reference beam, have been reported.^37^^,^^45^^,^^46^

In this paper, we propose the common-path off-axis configuration by employing a cube beam splitter, which divides the incoming object beam into two beams, and a pinhole is used to filter spatially one of the beams at its Fourier plane, serving a reference beam. The beam splitter-based common-path DHM systems have been reported previously,^44^^,^^47^ where the system^44^ is based on the self-referencing method, and in Ref. 47, two π phase-shifted holograms are recorded and these two holograms are processed by using an algorithm based on the subtraction of the two fields of view (FOVs) in order to eliminate the direct current (DC) term. These approaches are facing two major problems: (1) there is a possibility of overlapping of the object information at the image plane and may lead to generating twin images of the object and (2) if the first problem is overcome by utilizing only one half of the incident beam for object visualization, and another half is used for the reference beam, then, this process reduces the overall FOVs of the system by half. Moreover, in Ref. 47, since two holograms are recorded simultaneously by utilizing the half of the sensor area for the transmitted object beam from the beam splitter and another half area by the reflected object beam, which is π phase-shifted by the transmitted beam, it reduces the FOV of the system. Therefore, these problems can be overcome by spatially filtering one of the beams at its Fourier plane using a pinhole, obtained after passing through the beam splitter. Further, to retrieve the phase distribution in coherent DHM, we adopted a single-shot holographic method by utilizing principal component analysis (PCA)-based phase aberration compensation method,^48^ which enables the system for the investigation of fast-dynamic events. Therefore, only a single hologram is recorded in the presence of the object and processed to obtain the phase information. Further, both the proposed, coherent and incoherent, DHMs are integrated to develop a new configuration of the multimodal system, which could, indeed, have the capability to provide high-contrast functional imaging along with structural details of the biological specimen on a single platform. It is possible to extract important intrinsic biophysical parameters of the biological specimen from the proposed multimodal system.

## 2 Methodology

### 2.1 Three-Dimensional Fluorescence Imaging System

**Fig. 1:**
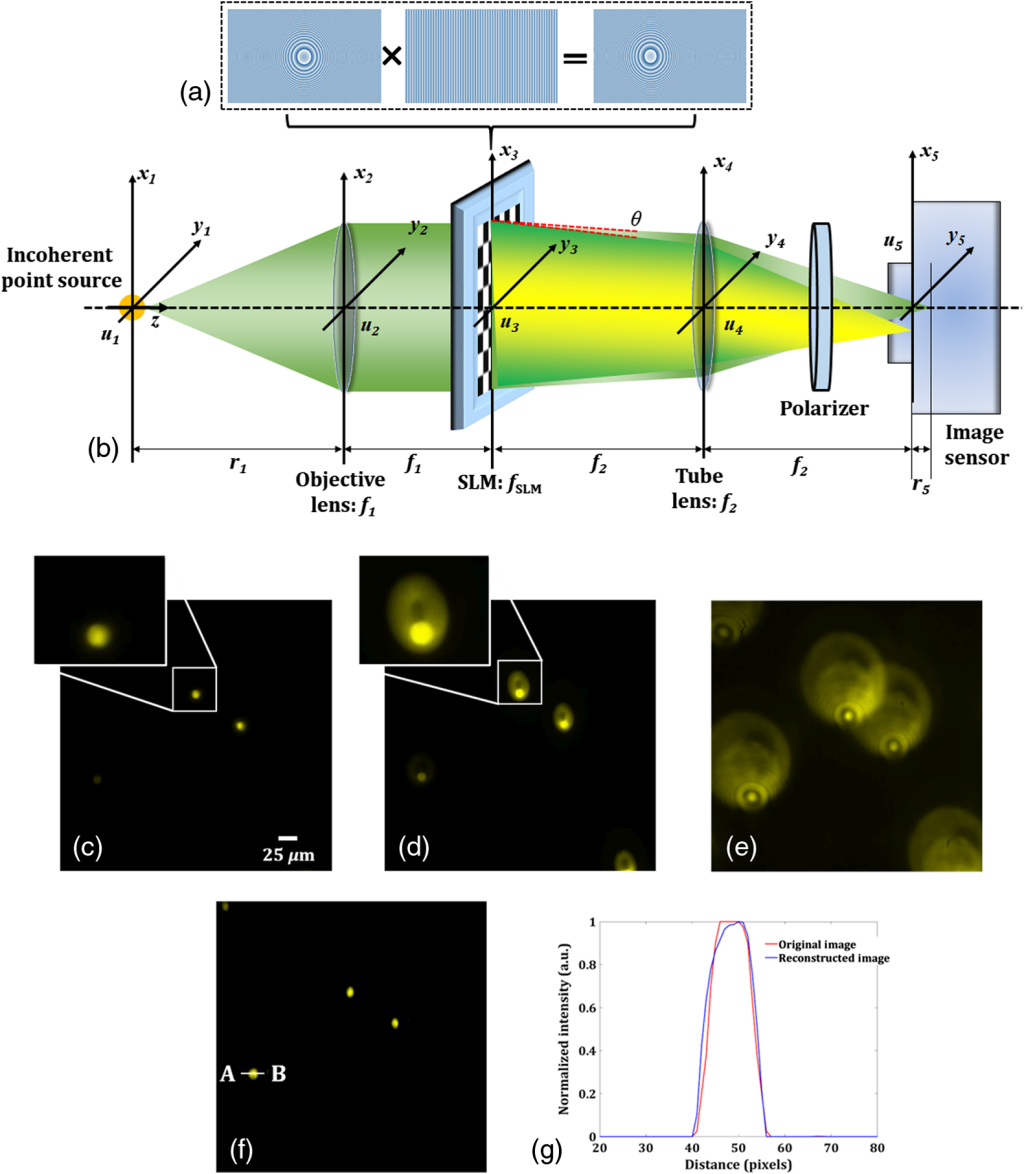
Incoherent digital holographic system for 3-D FI. (a) Schematic representation of the common-path off-axis digital holographic setup. (b) The phase pattern displayed on the SLM, (c) focused image of the fluorescent beads, and an enlarged view of one bead is also shown sideways, (d) image obtained when the pattern shown in Fig. 1(b) displayed on the SLM, and an enlarged view of one bead is also shown sideways, (e) recorded fluorescent hologram obtained by moving the sample in the z direction by −80  μm, (f) reconstructed image of the fluorescent beads, and (g) normalized intensity plot for the recorded and reconstructed selected fluorescence beads across the line AB, marked in (f).

The multimodal system comprises two DHMs for 3-D fluorescence and phase imaging. Figure 1(a) shows the schematic of the common-path off-axis fluorescent digital holographic system for 3-D FI recently developed by our group.^10^ This fluorescent microscope is accomplished by embedding a focusing lens with a diffraction grating, as shown in Fig. 1(b), onto a phase-mode spatial light modulator (SLM). This pattern allows splitting the incident fluorescent light from the object into two light waves with slightly different propagation directions in order to achieve off-axis interference. Figure 1(c) shows the focused image of the fluorescent beads of size ∼10.4  μm when no pattern is displayed on the SLM, and Fig. 1(d) depicts the two fluorescent lights: one unmodulated and another modulated by the pattern [shown in Fig. 1(b)] displayed on the SLM. These two wavefronts interfere at the image sensor plane and form a fluorescent digital hologram, as shown in Fig. 1(e), by the help of a linear polarizer, when the sample (fluorescent beads) is moved in the (±) z direction by a small distance, say −80  μm, as in this case.

If the radii of two wavefronts are denoted as $r_{m}$ and $r_{u}$ by setting the focal length of $f_{\text{SLM}}=f_{0}$ and infinity, respectively, the reconstructed distance from the image sensor plane is described as $z_{h}=r_{m}r_{u}/r_{m}-r_{u}$. From the recorded fluorescence hologram, the 3-D object information can be reconstructed by using the Fresnel propagation algorithm. Figure 1(f) shows the reconstructed image of the fluorescent beads, and the normalized intensity plot is shown in Fig. 1(g) for the recorded and reconstructed selected fluorescence beads across the line AB, marked in Fig. 1(f). The signal-to-noise ratio (SNR) for the recorded image (SNR=30.4445) and the reconstructed image (SNR=29.8611) are almost the same. This shows the good performance of the system.

### 2.2 Three-Dimensional Phase Imaging System

**Fig. 2:**
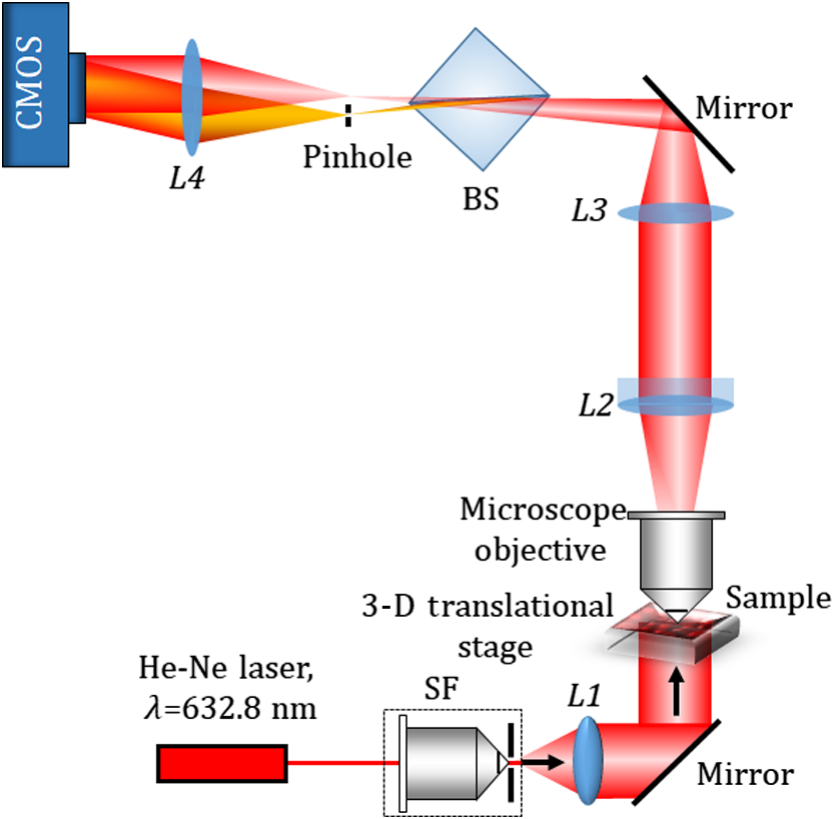
Schematic representation of the proposed common-path off-axis DHM setup. BS, Beam splitter; SF, spatial filter; L1 to L4, lenses.

The phase imaging is accomplished by a single-shot common-path off-axis DHM using a cube beam splitter. Figure 2 shows the schematic of the experimental setup of the proposed beam splitter-based common-path DHM. The collimated laser light (He-Ne laser, λ=632.8  nm) transilluminates the sample mounted on a motorized translational stage. The light transmitted through the sample is collected by a microscopic objective lens [40× magnification, numerical aperture (NA) = 0.65] and collimated by the lens L2 (focal length=100  mm). The collimated light is focused by L3 (focal length=150  mm) and allowed to pass through a cube beam splitter, BS, which divides the incident object beam into two beams. Since both the beams carry object information, their interference on the image sensor may bear the overlapping issue. To avoid the overlapping, one beam is spatially filtered at the Fourier plane by using a pinhole (50  μm, diameter), mounted on a 3-D translational stage. The pinhole acts as a spatial filter and is positioned in such a way that it blocks the higher frequency components and allows only the DC component of the beam to pass and converted into a spherical reference beam, in which all the object information is erased. After passing through a lens L4, the reference wave becomes a tilted plane wave. This is good for ideal off-axis holography. The orientation of the beam splitter is made in such a way that the two divided beams have sufficient lateral separation. The interference of the object beam and the reference beam is recorded by a CMOS camera (Sony Pregius IMX 249, sensor format: 1920×1200  pixels, pixel size of 5.86  μm).

## 3 Experiments and Results

### 3.1 Common-Path Digital Holographic Microscopy

**Fig. 3:**
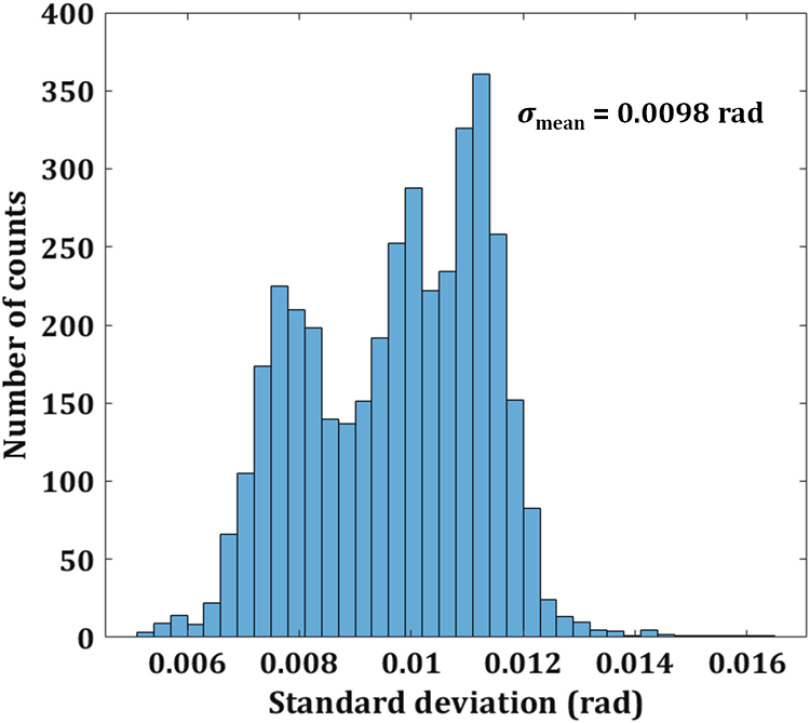
Temporal stability of the proposed setup. Histogram of the standard deviation between reconstructed phase distributions.

First, the performance of the single-shot common-path off-axis DHM is demonstrated. The proposed system, owing to its common-path configuration, shows high temporal stability compared to the conventional two-beam configuration of DHMs. To measure the temporal stability of the system, a series of holograms, without the presence of the object, are recorded at the rate of 40 frames per second, for 100 s without any vibration isolation. Then, the phase distributions are reconstructed numerically for all the 4000 holograms, and the phase difference distributions are calculated for all the frames by comparing the reconstructed phase distributions to that of the first recorded hologram. The standard deviation of the phase difference for 10,000 random pixel points in the same area of every phase difference distribution is calculated. Figure 3 shows the histogram of the standard deviation of the proposed setup indicating that a mean fluctuation is 0.0098 rad. On the other hand, the mean variation of phase is ∼0.2  rad for the traditional two-beam (e.g., Mach–Zehnder) interferometer.^3^ Therefore, the proposed setup shows improved temporal stability in comparison to its counterparts.

**Fig. 4:**
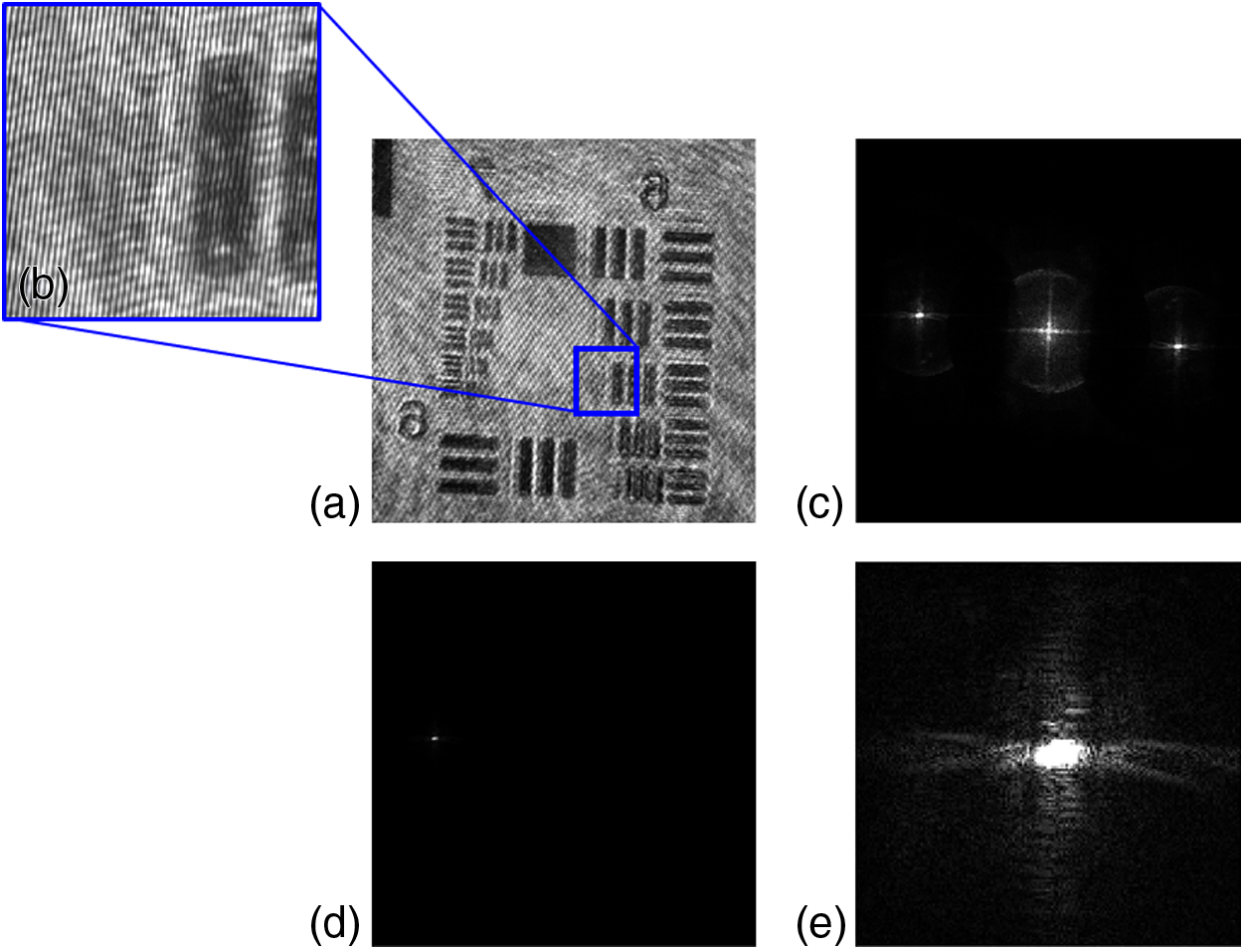
Common-path off-axis DHM results of USAF resolution chart: (a) recorded digital hologram, (b) the enlarged view of the selected region showing the fringes, (c) Fourier spectrum of the hologram, (d) filtered +1 order, and (e) +1 order centered (contrast adjusted).

**Fig. 5:**
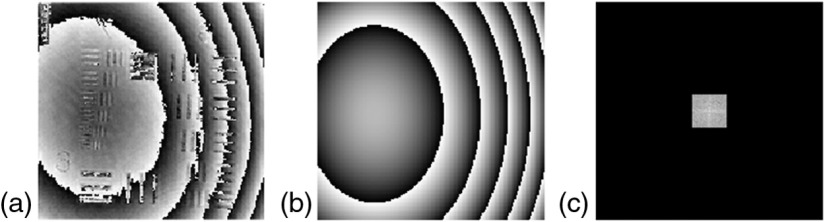
(a) Raw sampled phase, (b) conjugate phase extracted, and (c) compensated spectrum.

**Fig. 6:**
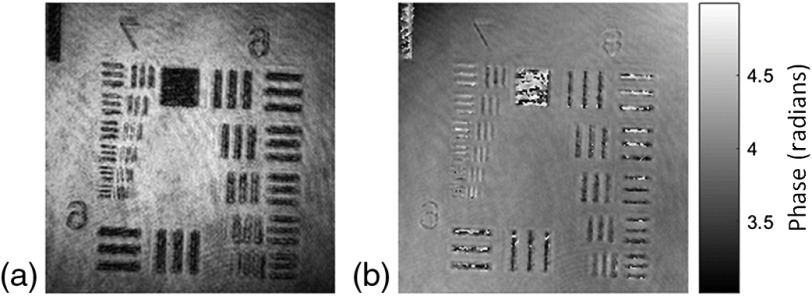
(a) The reconstructed intensity image corresponding to the recorded hologram shown in Fig. 4(a) and (b) unwrapped phase map.

Then the imaging capability of the proposed DHM is demonstrated by performing several experiments on objects, such as a United States Air Force (USAF) resolution chart, microsphere beads, and 3-D biological sample. Only a single hologram, in the presence of the object, is recorded and is further used to extract the object intensity and phase distribution. Figures 4–6 show the experimental results on the USAF resolution chart. Figures 4(a) and 4(b) show the recorded hologram and the enlarged view of the selected region of the recorded hologram, respectively. Figures 4(c)–4(e) show the Fourier spectrum of the recorded hologram, filtered +1 order, and +1 order centered, respectively. A spherical phase curvature is introduced in the object wave after passing through the microscopic objective and that must be compensated. The numerical phase aberration compensation method based on the PCA is utilized in order to accurately recover the phase information.^48^ This method is based on the decomposition of the phase map into a set of values of uncorrelated variables. These variables are called principal components and from the first principal component the aberration term is retrieved. The first principal component of the exponential term of the filtered hologram is estimated by using a singular value decomposition. Then the linear and the quadratic coefficients can be identified using least-squares fitting and their conjugate is multiplied with the filtered hologram in order to obtain aberration-free phase distribution. These steps involved extracting aberration-free phase distribution using the PCA method are demonstrated in Figs. 5(a)–5(c). Figure 5(a) shows the raw sampled phase distribution carrying the phase aberration. Figure 5(b) shows the obtained phase aberration distribution using the PCA method. The conjugate of this aberration term is multiplied with the sampled hologram [Fig. 5(a)] and results in the modification of the original region of the spectrum. The new modified spectrum is shown in Fig. 5(c). Then the numerical reconstruction process is performed to obtain the aberration-free phase distribution. Figures 6(a) and 6(b) show the retrieved intensity and phase distribution corresponding to the recorded hologram shown in Fig. 4(a). The PCA-based phase aberration compensation method does not require prior knowledge either of the object or of the setup and shows efficient performance with promising results.^48^

**Fig. 7:**
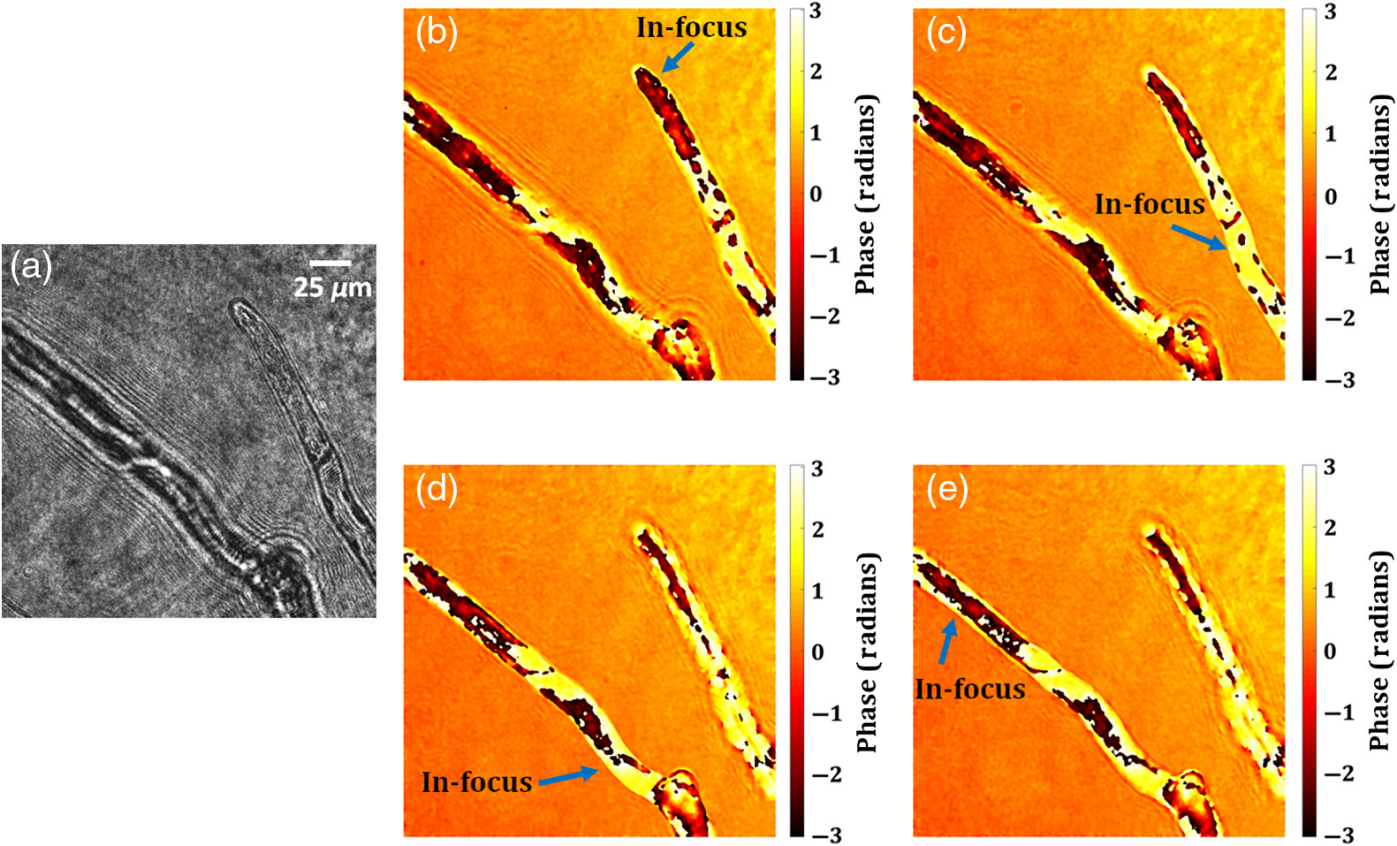
Common-path off-axis DHM experimental results of living plant cells of protonemata, the hypha-like structure of Physcomitrella: (a) digital hologram and (b)–(e) some of the retrieved wrapped phase maps at different in-focus planes (the in-focus region is indicated by the blue arrows). (Video 1, mp4, 6442 KB [URL: https://doi.org/10.1117/1.JBO.25.3.032010.1]).

In the next experiments, the capability of the proposed DHM system is demonstrated on a 3-D biological sample, the living plant cells. As the observation target, we used the moss *Physcomitrella patens* (Physcomitrella).^49^ In Physcomitrella, the genome is sequenced,^50^ the cell identity is clear,^49^ and the body size is compact, all of which make Physcomitrella one of the model organisms for modern biology. Therefore, Physcomitrella is suitable as the observation target using the proposed system. Figure 7 shows the experimental results of the proposed DHM on the living plant cells of protonemata, the hypha-like structure of Physcomitrella. The recorded hologram of the 3-D sample is shown in Figs. 7(a) and Figs. 7(b)–7(e) show the wrapped phase maps of the four different in-focus planes obtained from the recorded hologram, where blue arrows indicate the in-focus regions. Video 1 depicts the movie of the recovered in-focus phase imaging from one plane [corresponding to the in-focus plane of Fig. 7(b)] to another [to the in-focus plane of Fig. 7(e)].

### 3.2 Multimodal System

**Fig. 8:**
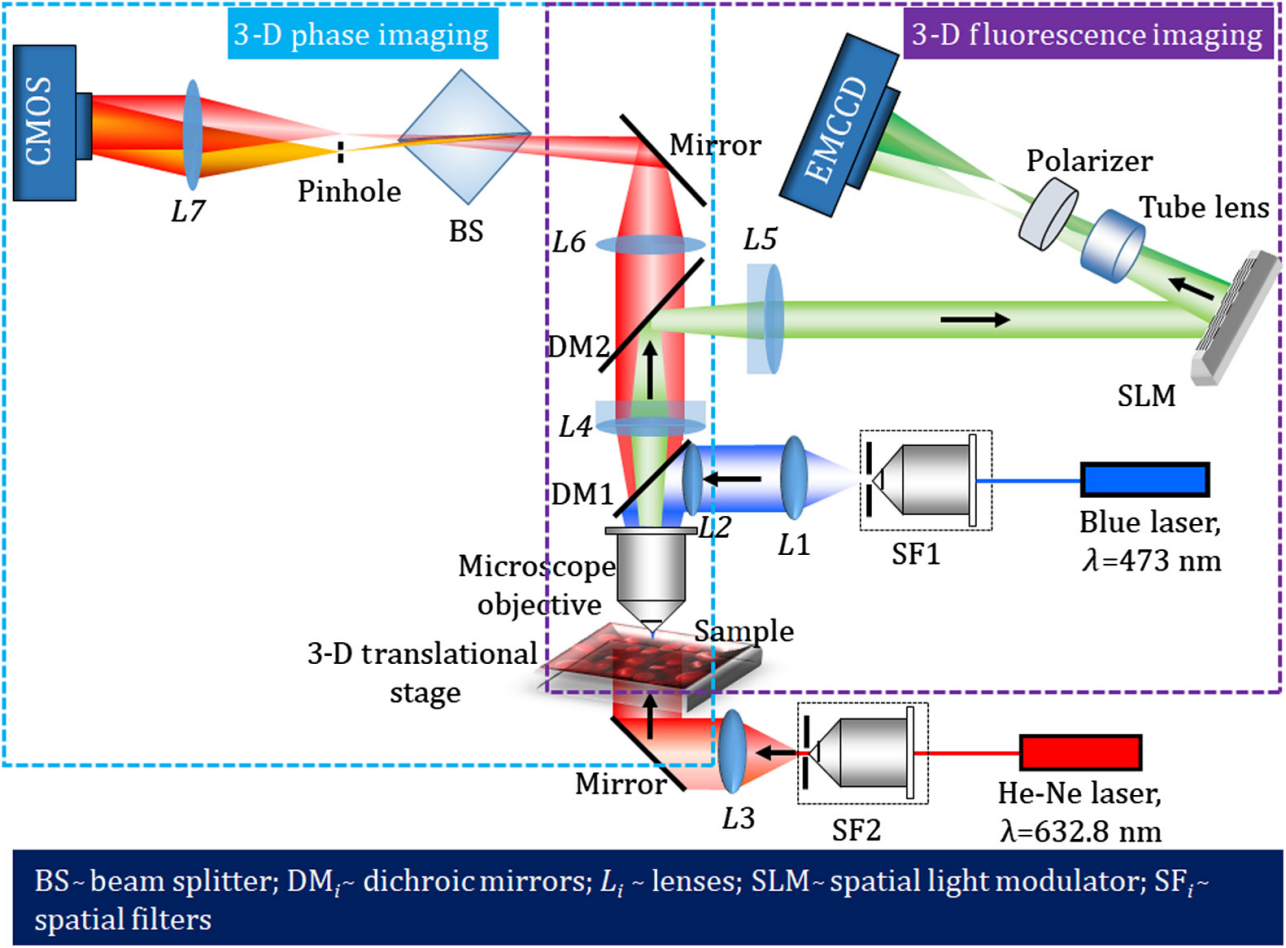
Optical schematic of the proposed multimodal system for the measurement of the 3-D fluorescence and the 3-D phase of the specimen.

In this section, we demonstrate the performance of the multimodal imaging system developed by combining the common-path off-axis DHM and the common-path off-axis FDHM, in order to retrieve simultaneously both the 3-D phase and 3-D fluorescence images. Figure 8 shows the schematic of the proposed multimodal system. Here, we use two image sensors for fluorescence and phase imaging. For the applications of biological samples, the optical power difference is too much to record both holograms by a single image sensor because the fluorescence light is too weak to avoid phototoxicity. However, two cameras create an image registration problem. In other applications, such as material sciences, a single image sensor is ideal for fabricating an integrated multimodal system. The performance of the proposed multimodal system is demonstrated by performing experiments on microsphere fluorescent beads and fluorescent protein-labeled living plant cells. A blue laser (wavelength, λ=473  nm) is used as a light source for the incoherent DHM system, to excite the fluorescent object used in the study. The laser beam is spatially filtered, collimated, and then reflected from a dichroic mirror (DM1, Thorlabs, DMLP490R) and entered into the objective lens (40× magnification, NA=0.65). The incident laser light excites the fluorescent object. First, an experiment is performed on microsphere fluorescent beads of size ∼10.4  μm. The beads, mounted on the 3-D translational stage, are excited by the collimated laser beam in an epi-illumination configuration. The fluorescent beads emit the yellow fluorescence with wavelengths ranging from 550 to 600 nm. This fluorescent light travels back through the objective lens, transmits through DM1, and is reflected from another dichroic mirror, DM2 (Thorlabs, DMLP605R). The Fourier transform of the object beam located at the focused plane of the objective lens is projected onto the plane of the phase-mode SLM (Holoeye pluto, 1920×1080  pixels, 8  μm pixel pitch, phase-only modulation) by a 4f relay system (L4→L5). Here, the back focal plane of the objective lens is imaged on the phase-mode SLM. A lens function with focal length $f_{\text{SLM}}=800\;\mathrm{mm}$ and a diffraction grating function with grating period $d_{h}=300\;\mu m$ were displayed onto the SLM, which generates two distinct wavefronts at respective angles of the incident beam. These two wavefronts are then imaged by a tube lens (focal length=200  mm) and allowed to interfere on to the faceplate of the electron multiplying charge-coupled device (EMCCD) sensor (Andor iXon 888, sensor format: 1024×1024  pixels, pixel size of 13  μm, sensor diagonal of 18.8 mm) and hence form a fluorescent digital hologram. A linear polarizer is placed before the EMCCD sensor in order to allow the interference of two beams. The volume size of the 3-D fluorescence system we demonstrated in the experiments is $330\times 330\times 100\;{\mu m}^{3}$. The axial size can be more extended. The axial measurable size will be determined by setting the required resolution to be reconstructed.

The proposed common-path off-axis DHM system for phase imaging uses a He-Ne laser (λ=632.8  nm) to transilluminate the microsphere fluorescent beads mounted on the translational stage. The collimated beam illuminates the microsphere beads and is subsequently magnified by the microscope objective and collimated using the lens L4. The object beam passes through both the DMs and again focused by using the lens L6. This object beam is divided into two beams by using the beam splitter, BS, in which one beam behaves as an object beam carrying the object information and the other beam is spatially filtered at its Fourier plane by using a pinhole of size 50  μm and serves the reference beam. The interference of the reference and object beams is recorded by a CMOS camera (Sony Pregius IMX 249, sensor format: 1920×1200  pixels, pixel size of 5.86  μm). The volume size of the fabricated phase imaging system is $280\times 176\times 80\;{\mu m}^{3}$. The axial size can also be more extended. The axial measurable size will be determined by setting the required resolution to be reconstructed.

**Fig. 9:**
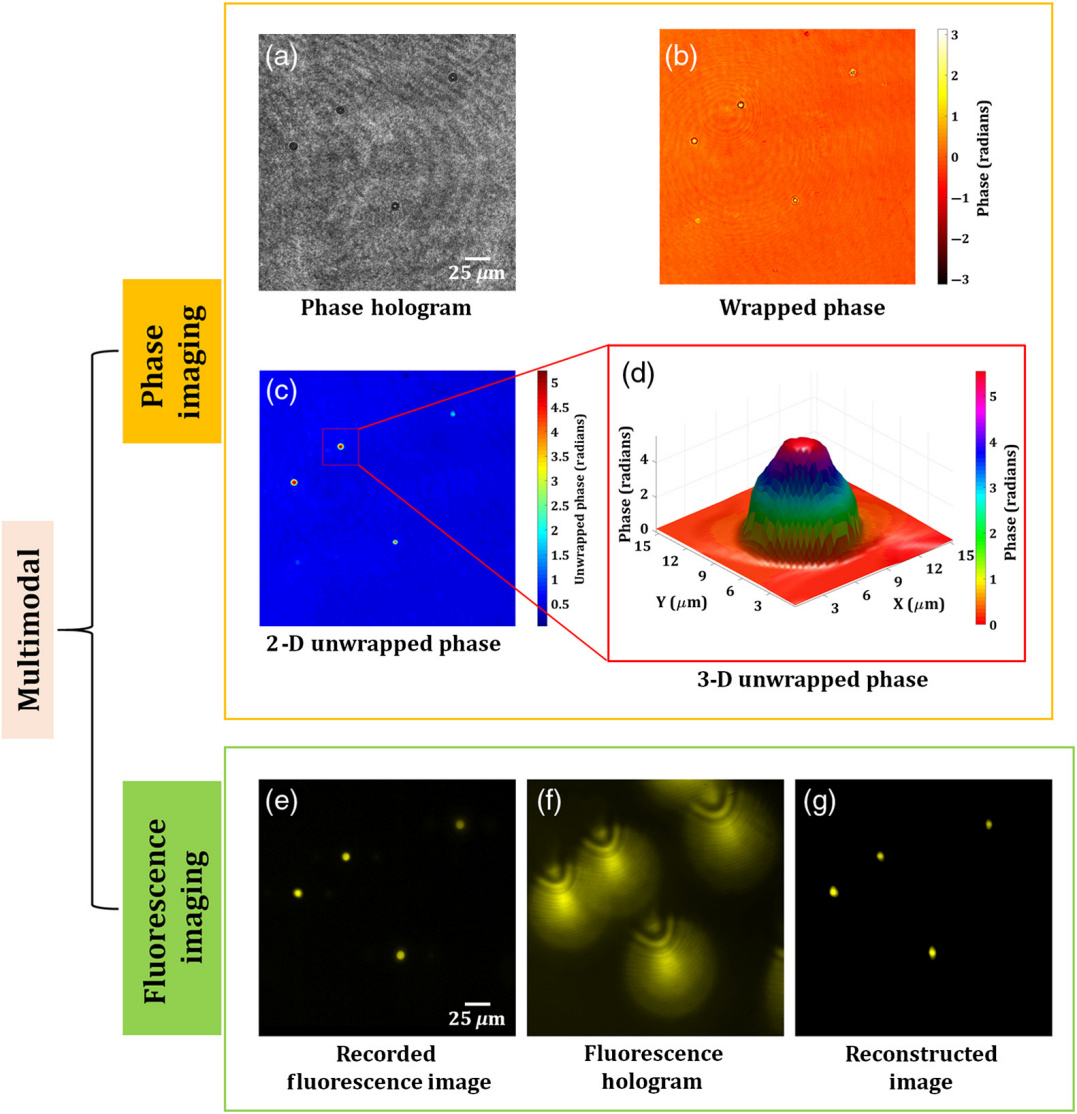
Experimental results of the multimodal system of microsphere beads. Phase imaging results: (a) phase hologram, (b) wrapped phase distribution, (c) 2-D unwrapped phase map, and (d) 3-D unwrapped phase distribution of a selected bead. FI results: (e) original focused image of the fluorescent beads, (f) fluorescent digital hologram obtained by moving the beads by 80  μm along the z direction, and (g) reconstructed image of the fluorescent beads.

Figure 9 shows the experimental results of the proposed multimodal system on microsphere fluorescent beads of size ∼10  μm. Figure 9(a) shows the phase hologram recorded by common-path off-axis DHM. From this single-phase hologram, the phase information is retrieved by the PCA-based phase aberration compensation method. Figures 9(b) and 9(c) show the retrieved wrapped and 2-D unwrapped phase maps, respectively, of the fluorescence beads. The 3-D phase distribution of a selected bead is shown in Fig. 9(d). Concurrently, the fluorescent beads are imaged by FDHM. Figure 9(e) shows the focused image of the microsphere fluorescent beads. Then, the stage is moved in the z direction by 80  μm, and a fluorescent digital hologram, as shown in Fig. 9(f), is recorded. The fluorescent hologram is recorded by projecting a lens function and a diffraction grating function on to the SLM. A bandpass filter centered at 575±12.5  nm was placed in front of an EMCCD sensor in order to improve the fringe visibility of the holograms. Figure 9(g) shows the reconstructed image of the fluorescent beads retrieved from the recorded digital fluorescent hologram by the Fresnel propagation algorithm at the reconstruction distance of 1009 mm.

In the next experiment, we experimentally demonstrate the 3-D fluorescence and 3-D phase imaging capability of the proposed multimodal system on living plant cells of Physcomitrella, where the “Citrine” yellow fluorescent protein gene (YFP)^51^ was inserted into a histone H3.3 locus (Pp3c18_14481^50^) (see Fig. S1 and Supplementary text in the Supplementary Material, for more details), resulting in strong expression of H3.3-YFP in the nuclei.

**Fig. 10:**
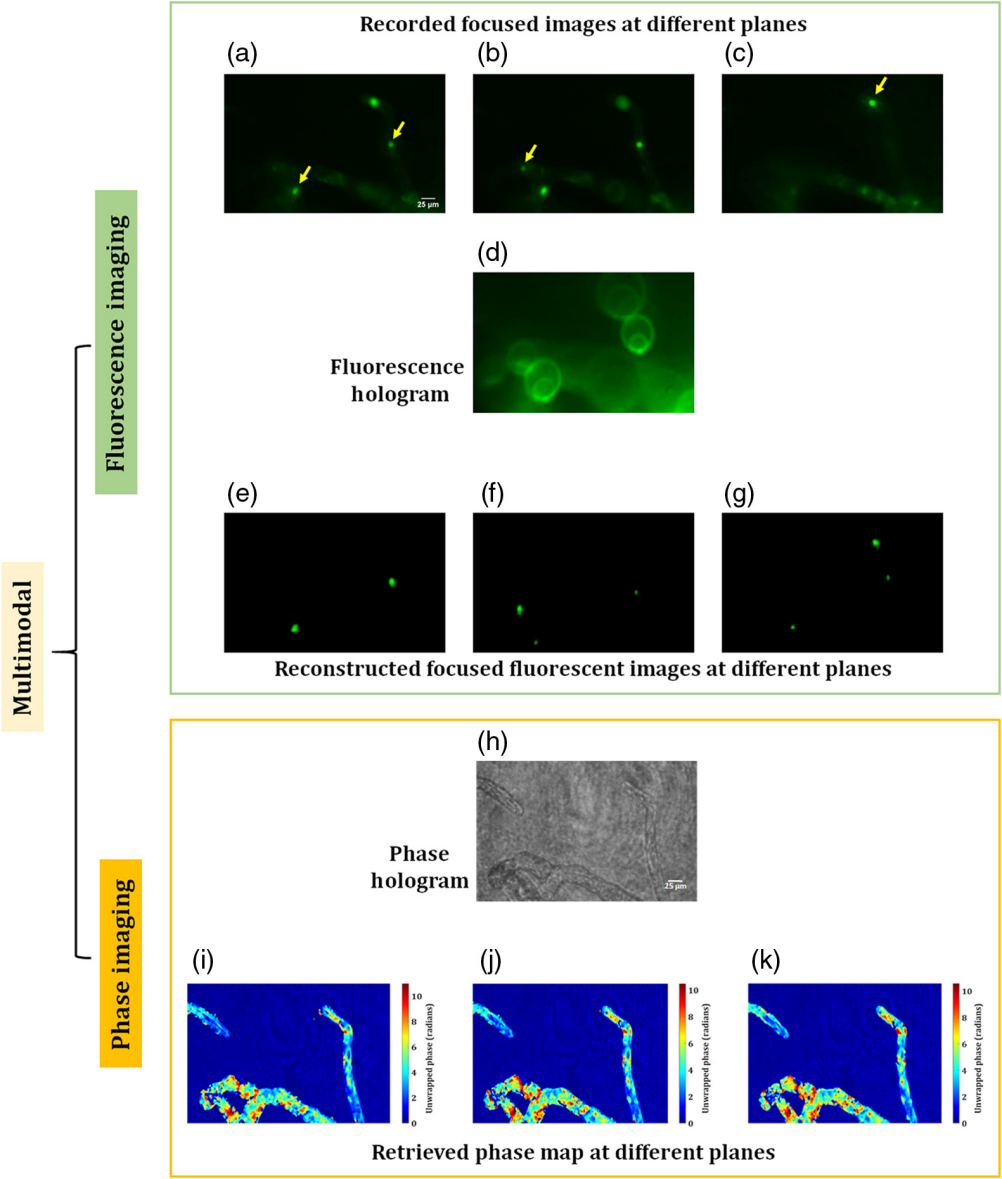
Experimental results of the multimodal system of living protonema cells of Physcomitrella. FI results: (a)–(c) original focused images of the nuclei (670×1024  pixels). The yellow arrow, in these figures, indicates the focused nuclei, (d) fluorescent digital hologram, and (e)–(g) reconstructed focused images of the nuclei correspond to Figs. (a)–(c). Phase imaging results: (h) phase hologram and (i)–(k) 2-D unwrapped phase maps corresponding to three focused planes.

Figures 10(a)–10(c) show the various focused fluorescence images of the nuclei of the living plant cells distributed in the 3-D space. The yellow arrow, in these figures, indicates the focused nuclei. The focused plane corresponding to Fig. 10(c) is moved by 60  μm in the z direction and a fluorescent digital hologram is recorded, as shown in Fig. 10(d), by projecting a lens function (with a focal length of 800 mm) and a diffraction grating function (with a grating period of 300  μm) on to the SLM. Figures 10(e)–10(g) show the reconstructed focused images corresponding to Figs. 10(a)–10(c), obtained at 660, 825, and 1030 mm, which correspond to 60, 70, and 80  μm, respectively, in the object space. The phase imaging results of the same plant cells are shown in Figs. 10(h)–10(k). Figure 10(h) shows the recorded phase hologram, and the unwrapped phase maps at three different focal planes are shown in Figs. 10(i)–10(k). Chloroplasts, which strongly delay the phase^52^ and thus appear as dark spots in Figs. 10(i)–10(k), are clearly imaged near the focal plane. Taken together, the proposed multimodal approach can retrieve the 3-D structure of the cells as well as the 3-D position of the fluorescent protein-labeled nuclei in living plant cells. These results show the high potential of the proposed system to observe the 3-D behavior of living cells in a single-shot measurement.

**Fig. 11:**
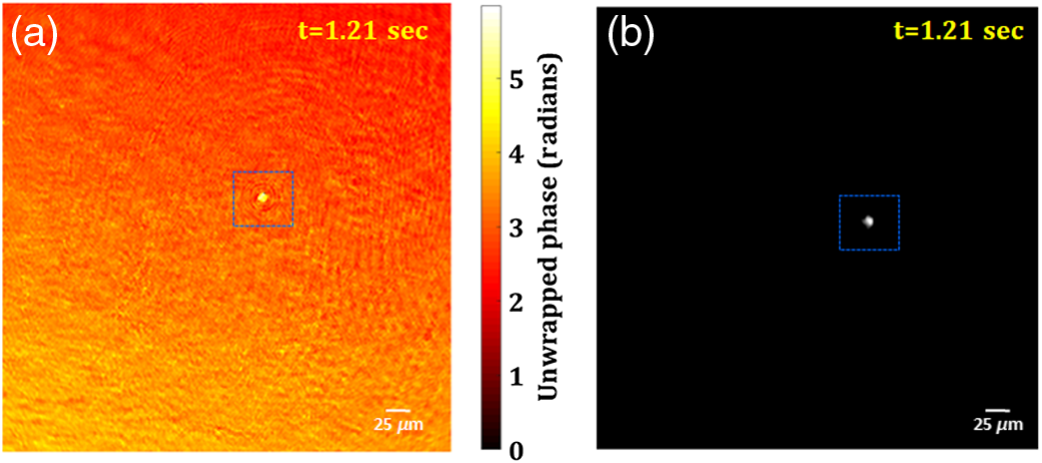
3-D live phase and FI results: (a) unwrapped phase map and (b) fluorescence image of the moving fluorescent bead at t=1.21  s. Also, see Video 2 (mp4, 5984 KB [URL: https://doi.org/10.1117/1.JBO.25.3.032010.2]) (retrieved unwrapped phase distribution) and Video 3 (mp4, 75 KB [URL: https://doi.org/10.1117/1.JBO.25.3.032010.3]) (reconstructed fluorescence image).

Further, the 3-D live fluorescence and phase imaging of the moving fluorescent beads in a volume is also demonstrated by the proposed multimodal system. Figures 11(a) and 11(b) show the retrieved unwrapped phase and reconstructed fluorescence image of the moving bead, respectively, at a time instant (t=1.21  s), obtained from the digital phase and fluorescence holograms recorded by the multimodal system. The movie of the retrieved unwrapped phase of the moving microsphere bead is shown in Video 2, whereas the movie of the reconstructed fluorescence images of the same bead is shown in Video 3.

## 4 Conclusion

First, a single-shot common-path off-axis coherent DHM based on a beam splitter is proposed. The common-path DHM is simple, compact, less vibration-sensitive, and provides high temporal phase stability of ∼0.0098  rad. Several experiments were performed in order to verify the imaging capability of the proposed highly stable DHM. Then, a new configuration of the multimodal system, by incorporating the proposed DHM in a combination of a single-shot common-path off-axis fluorescent digital holographic system, is demonstrated. The feasibility of the proposed multimodal system is exhibited by performing several experiments on fluorescent beads and living plant cells. The 3-D live fluorescence and phase imaging of the fluorescent beads is also demonstrated by the multimodal system efficiently. The experimental imaging results obtained by the proposed multimodal system corroborate the imaging capability of the system. The proposed multimodal system could be beneficial for the simultaneous measurement of the molecular-specific quantitative analysis and specific localized regions of the sample. Taken together, the system could be utilized for the comprehensive analysis of the living biological materials with specific molecular and biophysical dynamics, and physiological and pathological processes at a single platform. For example, the imaging of the dynamic phenomena, including cytoskeleton dynamics in the whole cells,^53^ neuronal activation in the brain and nerves,^54^ and morphogenetic flow during embryogenesis,^55^ could be studied. Therefore, such multimodality imaging systems could find an important role in a deeper understanding of cellular and developmental biology, monitoring of disease progression, and helpful in their improved diagnosis.
