# Supplementary material for: Common-path multimodal three-dimensional fluorescence and phase imaging system
Source: J Biomed Opt. 2020 Feb 6;25(3):032010. doi: 10.1117/1.JBO.25.3.032010 (PMC7003711; doi:10.1117/1.JBO.25.3.032010)
Supplement: Supplementary file 1 [file JBO_025_032010_SD001.pdf]

Common-path Multimodal 3D Fluorescence and Phase Imaging System

Manoj Kumar,[a,*] Xiangyu Quan,[a] Yasuhiro Awatsuji,[b] Chaoyang Cheng[c,d], Mitsuyasu Hasebe,[c,d,e] Yosuke Tamada,[c,e,f] and Osamu Matoba[a,]**

[a]Graduate School of System Informatics, Kobe University, Rokkodai 1-1, Nada, Kobe 657-8501, Japan
[b]Faculty of Electrical Engineering and Electronics, Kyoto Institute of Technology, Matsugasaki, Sakyo-ku, Kyoto 606-8585, Japan
[c]National Institute for Basic Biology, 38 Nishigonaka, Myodaiji, Okazaki 444-8585, Japan
[d]ERATO, JST, 38 Nishigonaka, Myodaiji, Okazaki 444-8585, Japan
[e]School of Life Science, SOKENDAI (The Graduate University for Advanced Studies), 38 Nishigonaka, Myodaiji, Okazaki 444-8585, Japan
[f]School of Engineering, Utsunomiya University, 7-1-2 Yoto, Utsunomiya 321-8585, Japan

Abstract: A new stable multimodal system is developed by combining two common-path digital holographic microscopes: coherent and incoherent, for simultaneous recording and retrieval of 3D phase and 3D fluorescence imaging, respectively, of a biological specimen. The 3D fluorescence imaging is realized by a single-shot common-path off-axis fluorescent digital holographic microscope (FDHM) developed recently by our group (Opt. Letters **43**(21) 5447-5450 (2018)). In addition, here we accomplish, the phase imaging by another single-shot, highly stable common-path off-axis digital holographic microscope (DHM) based on a beam splitter. In this DHM configuration, a beam splitter is used to divide the incoming object beam into two beams. One beam serves as the object beam carrying the useful information of the object under study, while another beam is spatially filtered at its Fourier plane by using a pinhole and it serves as a reference beam. This DHM setup, owing to a common-path geometry, is less vibration sensitive and compact, having a similar field of view but with high temporal phase stability in comparison to a two-beam Mach-Zehnder type DHM. The performance of the proposed common-path DHM and the multimodal system is verified by conducting various experiments on fluorescent microspheres and fluorescent protein-labeled living cells of the moss *Physcomitrella patens*. Moreover, the potential capability of the proposed multimodal system for 3D live fluorescence and phase imaging of the fluorescent beads is also demonstrated. The obtained experimental results corroborate the feasibility of the proposed multimodal system, and indicate its potential applications for the analysis of functional and structural behaviors of a biological specimen and enhancing the understanding of physiological mechanisms and various biological diseases.

Keywords: multi-modal, common-path configuration, digital holography, 3D fluorescence imaging, 3D phase imaging.

*Corresponding author, Email: manojklakra@gmail.com
** matoba@kobe-u.ac.jp

Supplemental Materials

Supplementary Methods

To prepare the targeting construct for the transformation of Physcomitrella Gransden 2004 strain (Fig. S1), the approximately 1-kb genomic region just before the stop codon of the target gene (5' fragment) was amplified with the primers 5'-GTGTCACGATGGCGCGTA-3' and 5'-TGCTCTTTCTCCTCTGATTCTTC-3', and the region including 3'-UTR of the target gene (3' fragment) was amplified with the primers 5'-GTTTGGGGTTCTTTTGAACCC-3' and 5'-GTAATTTGAGCATGTGGATTTC-3'. 5'- and 3' fragments were sequentially inserted into the pCTRN NPTII 2 vector (accession number AB697058[56]), which had been digested with *Eco*RV and *Sma*I, respectively. Linearization of the construct, polyethylene glycol-mediated transformation, PCR screening, and DNA gel blot analysis were performed as described previously.[57] H3.3-YFP #10 was used for the observation (see Fig. S1).

Supplementary Figure

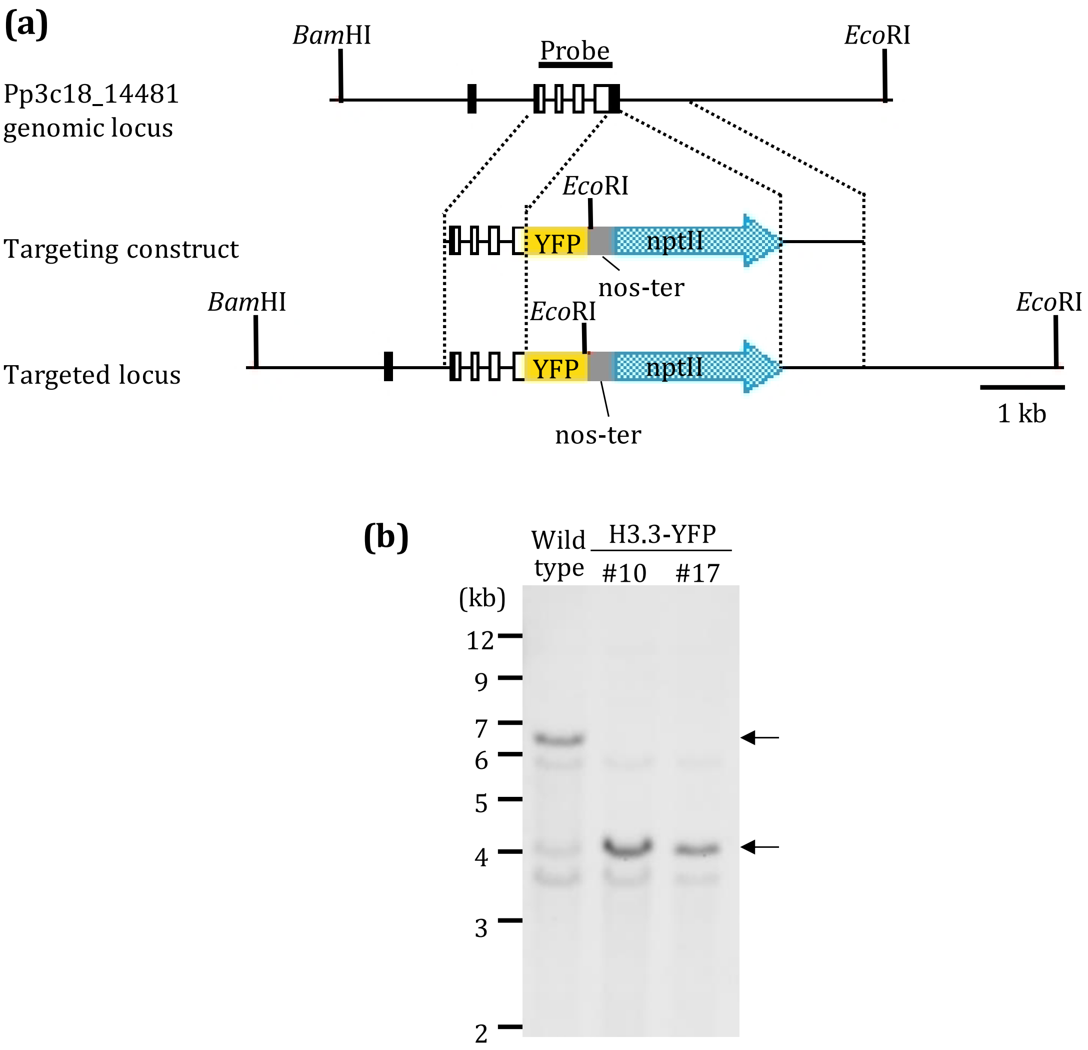

Fig. S1. Schematics of the construction of H3.3-YFP lines and DNA gel-blot analysis of the H3.3-YFP lines. (a) Schematics of the construction of H3.3-YFP lines. White and black boxes indicate coding sequence and untranslated regions on exons, respectively. Gray boxes and blue arrows denote the terminator of the nopaline synthase gene (nos-ter) and the neomycin phosphotransferase II expression cassette (nptII) of pCTRN NPTII 2,[56] respectively. A yellow box represents the *Citrine* yellow fluorescent protein gene (YFP),[51] which was inserted just before the stop codon of the H3.3 gene. (b) DNA gel-blot analysis of the H3.3-YFP lines. Genomic DNA of wild type and two independent H3.3-YFP lines was digested with *Bam*HI and *Eco*RI, blotted, and probed with the fragment indicated in (a). Extra bands detected in all lines are considered to be non-specific bands.
